# Predominant Biphenyl Dioxygenase From Legacy Polychlorinated Biphenyl (PCB)-Contaminated Soil Is a Part of Unusual Gene Cluster and Transforms Flavone and Flavanone

**DOI:** 10.3389/fmicb.2021.644708

**Published:** 2021-10-14

**Authors:** Jachym Suman, Michal Strejcek, Andrea Zubrova, Jan Capek, Jiri Wald, Klara Michalikova, Miluse Hradilova, Kamila Sredlova, Jaroslav Semerad, Tomas Cajthaml, Ondrej Uhlik

**Affiliations:** ^1^Department of Biochemistry and Microbiology, Faculty of Food and Biochemical Technology, University of Chemistry and Technology, Prague, Czechia; ^2^Institute of Microbiology, Academy of Sciences of the Czech Republic, Prague, Czechia; ^3^Institute of Molecular Genetics of the Czech Academy of Sciences, Prague, Czechia; ^4^Faculty of Science, Institute for Environmental Studies, Charles University, Prague, Czechia

**Keywords:** aromatic ring-hydroxylating dioxygenases, biphenyl dioxygenase, secondary plant metabolites, flavonoids, polychlorinated biphenyls, functional metagenomics

## Abstract

In this study, the diversity of *bphA* genes was assessed in a ^13^C-enriched metagenome upon stable isotope probing (SIP) of microbial populations in legacy PCB-contaminated soil with ^13^C-biphenyl (BP). In total, 13 *bphA* sequence variants (SVs) were identified in the final amplicon dataset. Of these, one SV comprised 59% of all sequences, and when it was translated into a protein sequence, it exhibited 87, 77.4, and 76.7% identity to its homologs from *Pseudomonas furukawaii* KF707, *Cupriavidus* sp. WS, and *Pseudomonas alcaliphila* B-367, respectively. This same BphA sequence also contained unusual amino acid residues, Alanine, Valine, and Serine in region III, which had been reported to be crucial for the substrate specificity of the corresponding biphenyl dioxygenase (BPDO), and was accordingly designated BphA_AVS. The DNA locus of 18 kbp containing the BphA_AVS-coding sequence retrieved from the metagenome was comprised of 16 ORFs and was most likely borne by *Paraburkholderia* sp. The BPDO corresponding to *bphAE_AVS* was cloned and heterologously expressed in *E. coli*, and its substrate specificity toward PCBs and a spectrum of flavonoids was assessed. Although depleting a rather narrow spectrum of PCB congeners, the efficient transformation of flavone and flavanone was demonstrated through dihydroxylation of the B-ring of the molecules. The homology-based functional assignment of the putative proteins encoded by the rest of ORFs in the AVS region suggests their potential involvement in the transformation of aromatic compounds, such as flavonoids. In conclusion, this study contributes to the body of information on the involvement of soil-borne BPDOs in the metabolism of flavonoid compounds, and our paper provides a more advanced context for understanding the interactions between plants, microbes and anthropogenic compounds in the soil.

## Introduction

Biphenyl dioxygenases (BPDOs) are type IV multicomponent aromatic ring-hydroxylating dioxygenases (ARHDs) (Kweon et al., 2008). They consist of a large and small subunit of a terminal dioxygenase (in the case of BPDO, designated BphA and BphE, also referred to as BphA1 and BphA2, respectively) and an electron transfer chain (Furukawa et al., 1989; Furukawa et al., 2004; Kweon et al., 2008). ARHDs play a crucial role in nature, being used by many aerobic bacteria for the activation of a thermodynamically stable benzene ring for its further fission and catabolism (Mason and Cammack, 1992). BPDOs, similarly to other ring hydroxylating dioxygenases, exhibit broad substrate specificity. For instance, not only do BPDOs oxidize biphenyl (BP) but also molecules with a similar structure, such as anthropogenic halogenated BPs, including polychlorinated biphenyls (PCBs) and diphenyl ethers (Furukawa et al., 1989; Sylvestre et al., 1996; Furukawa, 2000; Pieper, 2005; Robrock et al., 2011).

Although PCBs are not commonly present in soils, PCB-degrading bacteria are ubiquitous in soils and sediments (Focht, 1995). Additionally, efficient PCB degraders have been isolated from termite guts where their lignin-rich diet is decomposed (Chung et al., 1994). Consequently, lignin-derived phenolics have been proposed as evolutionary original substrates of PCB-degrading enzymes (Focht, 1995; Furukawa et al., 2004). More recently, Sylvestre and colleagues postulated that the BP degradation pathway in soil bacteria may have evolved to serve different ecological functions (Pham and Sylvestre, 2013), including the metabolism of secondary plant metabolites (SPMs) (Toussaint et al., 2012; Pham et al., 2015). It is hypothesized that SPMs, being a wide array of compounds, including flavonoids (Singer et al., 2003), evolved to assist the plant in tolerating frost, storing nutrients, reinforcing structurally, signaling to mutualists, as well as acting as allelopathic chemicals and chemicals protecting the plant from herbivory (Rausher, 2001). Because insects and other herbivores are continually developing mechanisms of resistance to SPMs, plants are driven to modify and develop new mechanisms of protection, including the modification of SPMs. These changes in plant SPM content and composition in turn affect soil microbial communities, which has resulted in the establishment of enzymes with broad substrate specificity in soil bacteria (Singer et al., 2003); this then leads to the hypothesis that enzymes originally evolved for the degradation, transformation and/or detoxification of SPMs are also fortuitously involved in the degradation of anthropogenic pollutants. However, there is still a severe lack of data on how SPMs are linked with biodegradative broad-substrate-specificity enzymes, including BPDOs.

The affinity between the substrate and the BPDO enzyme is determined by amino acid (AA) residues in key positions of the large subunit of the terminal dioxygenase. Mondello et al. (1997) identified four sequence regions of the C-terminal portion of BphA from *Paraburkholderia xenovorans* LB400 (BphA_LB400) which have a direct impact on the catabolic properties of the enzyme. Region III, a consecutive span of seven amino acids (Tyr335-Phe-Asn-Asn-Ile-Arg-Ile341 in the wild-type BphA_LB400), was found to have the largest influence on substrate specificity and regioselectivity (Vézina et al., 2008), with residues Tyr335 and Phe336 impacting on substrate binding and orientation (Barriault et al., 2004) and residues Asn338 or Ile341 on catalytic activity (Mohammadi and Sylvestre, 2005). More recently, the sequence of BPDO BphA_II__9_, a BphA_LB400-derivative whose region III was engineered toward more efficient PCB transformation (Dhindwal et al., 2016), was analyzed by Zhu et al. (2020) in order to reveal the contribution of the AA residues of this region and others in the sequence to its catalytic efficiency. The authors concluded that the residues Gly335, Asn337, Thr338, and Ile339 within region III together with Asp230 facilitate the catalysis of both BP and 4, 4′-diCB.

The majority of data on BPDOs accumulated to date have described BPDOs in bacterial isolates. However, more recent studies (Iwai et al., 2010; Strejcek et al., 2015) on BPDOs in environmental matrices have shown an extensive diversity of environmental BphA, sometimes differing in the AA patterns common for region III in the isolates (Sul et al., 2009; Strejcek et al., 2015). Nevertheless, there is an immense lack of knowledge about the function of such diverse BPDOs, and especially about their substrate specificity, which would enable predictions of the role of these BPDOs in the transformation of various aromatic substances present in the environment. With this in mind, the objective of this study was to characterize a *bphA*-coding sequence which was isolated from ^13^C-labeled DNA fractions after stable isotope probing (SIP) with ^13^C-BP performed in legacy PCB-contaminated soil (Uhlik et al., 2012). Due to an unusual AA pattern in region III of its predicted protein sequence, namely, Ala333, Val335, and Ser340, we designated the sequence BphA_AVS and aimed to reveal its presumably unusual substrate specificity and thus predict its function in the soil environment. For this purpose, we elucidated the genomic context of the *bphA_AVS* sequence and experimentally revealed the substrate specificity of the corresponding BPDO through heterologous expression in *E. coli*. The transformation of a Delor 103-contained spectrum of PCB congeners was investigated along with a series of flavonoids as compounds of plant origin widely found in soils and exhibiting structural homology with BP/PCBs (Singer, 2006).

## Materials and Methods

### Amplicon Sequencing and Processing of *bphA* Genes

The *bphA* genes were amplified from the ^13^C-enriched metagenome isolated from legacy PCB-contaminated soil after SIP with ^13^C-BP (14-day incubation time), as was described earlier (Uhlik et al., 2012). The amplicons were generated by PCR with primers adapted from Iwai et al. (2010), which were fused with barcode sequences (only forward primer) and sequencing adapters (454 Sequencing Application Brief No. 001-2009, Roche). PCR was performed in 20-μl reactions containing 0.2 mM dNTPs (Finnzymes, Finland), 0.2 μM primers (Generi Biotech, Czechia), 0.1 mg.ml^–1^ bovine serum albumin (New England BioLabs, Great Britain), 0.4 U of Phusion Hot Start II DNA Polymerase (Finnzymes, Finland) in the corresponding buffer, and template DNA (10–50 ng). The cycling conditions were as follows: 98°C for 3 min, 35 cycles of 98°C for 10 s, 56°C for 30 s, and 72°C for 30 s with final extension at 72°C for 10 min. The resulting PCR products were re-amplified in the 8-cycle reconditioning step (Thompson et al., 2002) using 0.5 μl of the first PCR product, 0.2 mM dNTPs, 0.2 μM primers, 0.1 mg.ml^–1^ bovine serum albumin and 1 U of Phusion Hot Start II DNA Polymerase in the corresponding buffer. Reconditioning PCR products were purified using AMPure XP Beads (Agencourt, Beckman Coulter, Brea, CA, United States) following the manufacturer’s instructions and pooled together for sequencing. Pyrosequencing was performed unidirectionally from the forward primer using a GS FLX + system with Titanium reagents (Roche, Germany) as described elsewhere (Polivkova et al., 2018). An analogous procedure was applied to total community DNA, which was isolated prior to the construction of SIP microcosms (Uhlik et al., 2012).

Sequence data were translated from the SFF to FASTQ format using the sff2fastq (v0.9.2^1^) utility. The sample barcode-forward primer and reverse primer sequences were trimmed off from the reads using Cutadapt (v2.10) (Martin, 2011) in linked adaptor mode (-g). The reads with untrimmed barcode-forward primer sequences and those shorter than 400 bp were discarded. The read error correction was performed using the package DADA2 (v1.12.1) (Callahan et al., 2016) in the R environment (R Core Team, 2017) with default settings except that the reads were truncated to 400 bp. Chimeric sequences were detected with the “pooled” method. To correct potential frameshifts remaining in the data, the nucleotide sequences were subjected to a frameshift-aware DIAMOND blastx search (v0.9.30) (Buchfink et al., 2015) against RefSeq bacterial proteins (downloaded 02/19/2020) (O’Leary et al., 2016) with subsequent frameshift-corrected sequence export as described by Arumugam et al. (2019). Using the DIAMOND blastx results, sequences with a top hit not relevant to the terms “Rieske domain” or “dioxygenase“ were removed from the dataset as non-specific targets. Finally, as the frameshift correction approach left “n” or “nn” in the sequences that represented deletion or insertion, respectively, and since all the frameshifts were localized in homopolymer regions, the inserted “n/nn” were corrected accordingly, i.e., changing “n” to a nucleotide from the homopolymer or removing “nn” together with one extra nucleotide from the homopolymer. This final set of corrected amplicon sequences will be further referred to as sequence variants (SVs).

### Sequencing of the ^13^C-Metagenome and Recovery of *bphA_AVS* and the Surrounding Region

Prior to shotgun sequencing, the ^13^C-metagenome was amplified using a REPLI-g Mini Kit (Qiagen) according to the manufacturer’s recommendations, in a 50 μl reaction with a DNA input amount of 0.3 ng and a reaction time of 8 h. Upon amplification, DNA was precipitated with 70% ethanol and 0.3 M sodium acetate at *−*20°C for 16 h. DNA was harvested by centrifugation (20,000 × *g*, 4°C, 20 min), washed twice with 70% ethanol, and finally resuspended in 50 μl of molecular biology-grade water. The amplified DNA was fragmented with a Bioruptor NextGen Sonication System (Diagenode) for 30 cycles with high performance set up to achieve 300–400 bp-long inserts. Fragment length distribution was controlled with an Agilent Bioanalyzer 2100 (High Sensitivity DNA chip). The sequencing library was prepared from an input amount of 82 ng of fragmented DNA using a NEBNext Ultra DNA Library Prep Kit for Illumina (New England BioLabs) with size selection conditions adjusted to the final library size in the range of 400–500 bp. Illumina Index Primer 11 was used for barcoding the sample. Final library PCR amplification included 9 cycles, and the resultant product was cleaned up with AMPure XP Beads (Agencourt). Library length profile and concentration were controlled with Agilent Bioanalyzer (High Sensitivity DNA chip) and Qubit 2.0 Fluorimeter (Life Technologies, Carlsbad, CA, United States), respectively. The library was sequenced in a NextSeq 500 Illumina platform (NextSeq Control Software 4.0), 2 × 150 bp paired-end mode using a High-Output v2.1 (300 cycles) Kit with loading molarity 1.8 pM, including 1% PhiX control.

Raw FASTQ files were processed with BBtools (v38.70^2^ as follows: (1) optical duplicates were removed with *clumpify.sh dedupe optical*; (2) low-quality regions were removed with *filterbytile.sh*; (3) reads with low average quality as well as adapters were removed with *bbduk.sh ktrim* = *r k* = *23 mink* = *11 hdist* = *1 tbo tpe minlen* = *100 ref* = *adapters ftm* = *5 ordered maq* = *18 maxns* = *0*; (4) synthetic artifacts were removed using *bbduk.sh k* = *31 ref* = *artifacts,phix ordered cardinality*; (5) read coverage was normalized using *bbnorm.sh target* = *1000*; (6) paired-end reads were merged with the use of *bbmerge-auto.sh strict k* = *93 extend2* = *80 rem ordered*; and (7) unmerged reads were once more filtered using *bbduk.sh qtrim* = *r trimq* = *10 minlen* = *100 ordered*. Contigs were assembled and scaffolded with metaSPAdes (v3.13.1) (Nurk et al., 2017) using both merged and unmerged reads.

The scaffold bearing *bphA_AVS* was identified by searching against the *bphA_AVS* amplicon SV in the assembly output using blastn (BLAST + v2.9.0) (Camacho et al., 2009). Genomic features were predicted and annotated using Prokka (v1.14.6) (Seemann, 2014). The putative functions of deduced proteins encoded by ORFs within the genomic proximity of *bphA_AVS*, herein referred to as the AVS region, were assessed manually by a blastp search against the UniProtKB/Swiss-Prot (The UniProt Consortium, 2018) and RefSeq database (O’Leary et al., 2016), combined with the literature review.

### Taxonomic Classification of the Putative bphA_AVS-Bearing Organism

To determine the putative organism of origin of the *bphA_AVS* sequence, ORFs localized on the scaffold were searched against the NCBI RefSeq protein database (downloaded 02/19/2020) (O’Leary et al., 2016) using DIAMOND (v0.9.30) (Buchfink et al., 2015). For each query, the top hit subject’s taxonomy was extracted. In addition to NCBI taxonomy, the Genome Taxonomy Database’s (GTDB) (Parks et al., 2020) rank normalized classification was derived as well. The number of top hits and their average protein identity were used to assign the putative taxonomy of the *bphA_AVS*-host organism.

### Phylogeny Reconstruction of ARHD Large Subunits

In order to assess the phylogenetic relationship of BphA proteins corresponding to the *bphA* amplicon SVs, their deduced AA sequences were used for a homology search through the BLASTp (BLAST + v2.9.0) algorithm against the RefSeq database (O’Leary et al., 2016). The top hits, together with the set of exemplary BphA large subunit sequences whose functional characterization has been reported to date, were cropped to the length of *bphA* SVs (corresponding to the positions Gln226-Val358 in BphA_LB400). The cropped sequences were aligned using T-Coffee (mode Expresso) (Di Tommaso et al., 2011), and their phylogeny was reconstructed using the Maximum Likelihood method and JTT matrix-based model (Jones et al., 1992) within the MEGA X environment (5 categories for the discrete gamma distribution of rates among sites, some sites allowed to be evolutionarily invariable, all sites were used for the reconstruction) (Kumar et al., 2018). To reveal the phylogeny of the complete BphA_AVS mined from the metagenome in the context of Rieske-type ARHDs functionally characterized to date, the set of full-length sequences of ARHD large subunits was created based on a manual literature research. The AA sequences were aligned, and their phylogeny was reconstructed in the same way as for amplicon SVs. The alignment of BphA sequences was visualized using BOXSHADE version 3.21.

### Growth Media, Plasmids, Strains, and Growth Conditions

Lysogeny Broth (LB, 10 g/l Tryptone, 5 g/l yeast extract, 10 g/l NaCl) was used for all cultivations in this study. *Escherichia coli* DH11S (Lin et al., 1992) was used for both vector construction and gene expression. When needed, the antibiotics ampicillin, kanamycin, or their combination were added to the media to final concentrations of 150 and 50 μg.ml**^–^**^1^, respectively. The following plasmids were used: (i) for the expression of BPDO mined from the metagenome, pQE-31 plasmid was employed (ampicillin selection; Qiagen), (ii) for the complementation of BphF and BphG activities necessary for the BPDO function, pYH31-bphFGBC_LB400 plasmid was used, bearing the bphFGBC gene cluster from *Par. xenovorans* LB400 [kanamycin selection; original name pYH31[LB400-*bphFGBC*], kindly provided by prof. Michel Sylvestre (Chebrou et al., 1999)].

### BPDO_AVS Expression Vector Construction

The expression plasmid using the pQE-31 (Qiagen) backbone was constructed employing an In-Fusion^®^ HD Cloning Kit (Clontech Laboratories). The target cluster bphAE_AVS was amplified from the *^*13*^*C-enriched DNA using HotStart ReadyMix (KAPA Biosystems) employing the primers AVS_F (5′-GAGGAGAAATTAACTATGAGTACGACGATGAAGGAA) and AVS_R (5′-AACAGGAGTCCAAGCTTAGAAGAACATG CTGAGGTTGTTCG; the 15 bp overlap sequences on the 5′-ends enabling cloning by the In-Fusion^®^ HD Cloning Kit are underlined). The backbone of pQE31 was inverse PCR-amplified using the primers pQE_F (5′- GCTTGGACTCCTGTTGATAGATCC) and pQE_R (5′- AGTTAATTTCTCCTCTTTAATGAATTCTGTGTG) (Supplementary Figure 1). Both fragments were then fused using the In-Fusion^®^ HD Cloning Kit, yielding the plasmid pQE31-bphAE_AVS, in which the genes bphAE_AVS are located 8 bp downstream from the pQE-31 *E. coli*-consensual ribosome binding site; the DNA span encoding for the His-tag was omitted from the original plasmid sequence. The nucleotide sequence of the insert was verified by Sanger sequencing. The resulting plasmid, pQE31-bphAE_AVS, was used for the transformation of *E. coli* DH11S competent cells already hosting pYH31-bphFGBC_LB400. Upon their selection in the presence of a combination of ampicillin and kanamycin, grown colonies were PCR-screened for the presence of both plasmids and used for BPDO-heterologous expression assays.

### Heterologous Expression of BPDO_AVS and PCB/flavonoid Depletion Assay

**FIGURE 1:**
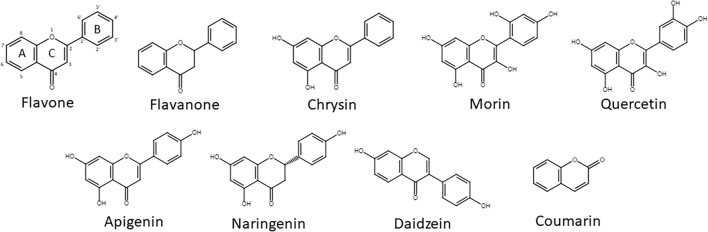
SPMs used in this study. The numbering of atoms is shown, as well as the designation of the A-, B-, and C-rings in the flavone structure.

In order to assess the PCB/flavonoid transformation capability of the BPDO_AVS, the cells of *E. coli* DH11S were employed, bearing pYH31-bphFGBC_LB400 either in combination with the plasmid pQE31-bphAE_AVS or empty pQE-31 (control cells in terms of the presence of BPDO_AVS), following a modified procedure of San-Miguel et al. (2013). A single colony picked from a plate was grown overnight in LB medium supplemented with ampicillin and kanamycin. This culture was then used for the inoculation of fresh LB medium with antibiotics. The cells were cultivated at 37°C on a rotary shaker (220 rpm) until OD_600_ reached a value of ca 0.5 (ca 5 h). The culture was then cooled down on ice and IPTG was added to a final concentration of 0.3 mM. The culture was then cultivated for ca 20 h at 16°C/130 rpm. Upon cultivation, the cells were harvested (5,000 × g/5 min), washed twice with cold mineral salt solution (MSS) (Uhlik et al., 2009; Wald et al., 2015) and eventually resuspended in MSS to achieve a final OD_600_ = 5. Prior to further assays, the activity of BPDO in *E. coli* cells was verified by adding 10 μl of 50 mM ethanolic BP solution to a 1 ml-aliquot of the washed cells suspension – in the presence of the active BPDO holoenzyme as well as BphB and BphC proteins, this amendment resulted in the quick evolution of the bright yellow BP-transformation intermediate 2-hydroxy-6-oxo-6-phenyl-2,4-hexadienoic acid (HOPDA). The cells were then subjected to a modified resting cell assay as described in Vergani et al. (2019) and Garrido-Sanz et al. (2020). Briefly, 5 ml aliquots of the cell suspension were distributed to 119 ml glass microcosms, then the substrate was added, and the microcosms were hermetically sealed and cultivated for 24 h at 28°C/130 rpm. For PCB depletion assessment, 3 μl of 10 mg/ml of Delor 103 solution in acetone was added. SPMs (flavone, flavanone, daidzein, quercetin, chrysin, naringenin, apigenin, morin, and coumarin, Figure 1) or BP (all purchased from MilliporeSigma, DE, except daidzein that was provided by Cayman Chemical, United States) were added in the form of an ethanolic solution so that the final concentration was 25 μM. Upon incubation with the substrates, the microcosms were stored at −80°C until further analyses. All microcosms were set up in quadruplicates. For the SPMs, non-biotic control, i.e., microcosms containing only MSS with added substrate with no cells, were also prepared and analyzed in order to assess non-specific transformation/depletion during the experiment.

### Depletion of Biphenyl, Flavone, and Flavanone Using Crude Whole-Cell Extract

In order to compare the depletion rates of biphenyl, flavone and flavanone, the BPDO_AVS was expressed in *E. coli* cells as described above. The resulting *E. coli* cell suspension with active BPDO_AVS was concentrated in MSS by centrifugation to the theoretical OD_600_ = 20 and disrupted using a One Shot cell disruptor (pressure 1.95 k Bar; Constant Systems Ltd.). Upon disruption, cell debris was removed by centrifugation (20,000 × *g*, 10 min) and microfiltration (0.45 μm). The crude whole-cell extract was then distributed by 50 μl into 1.2 ml glass vials, followed by the addition of 2 μl of 0.5 mM substrate solution in DMSO corresponding to a final concentration of 20 μM. The reactions were terminated by the addition of an equal volume of methanol and the samples were stored at −80°C until further analyses. The crude cell extract from *E. coli* cells bearing empty pQE-31 plus pYH31-bphFGBC_LB400 was used as a control of non-enzymatic substrate depletion.

### Analysis of PCB and SPM Depletion and Resultant Transformation Products

PCB depletion in microcosms was analyzed using gas chromatography-mass spectrometry (GC-MS) as described in Vergani et al. (2019). The individual PCB congeners were quantified using external calibration. The calibration curves were constructed in the concentration range of 0.001 to 0.750 μg/ml using the IADN PCB Congener Set (AccuStandard, United States) and several other pure congeners obtained from AccuStandard, Merck (Germany) and Dr. Ehrenstorfer (Germany). The regression coefficients of the calibration curves were >0.998. The depletion rates of individual congeners or SPMs were expressed as R = (SPM content in the presence of a respective strain)/(SPM content in the abiotic control sample). The calculation of standard deviations (SD) of the resulting *R* values was based on the propagation of errors according to Tellinghuisen (2001).

The depletion of SPMs was assessed by liquid chromatography–mass spectrometry (LC–MS). The cell suspension after the resting cell assay was sonicated for 15 min (Ecoson, Slovakia) and centrifuged at 2,000 × *g* for 10 min (Hettich EBA200, Germany). The supernatant was diluted appropriately with 50% methanol and analyzed with LC-MS. Analysis of SPMs in the extract was performed using an Agilent 1260 Infinity II liquid chromatograph coupled to an Agilent 6470 LC/TQ mass spectrometer (target analysis) or a high-resolution Agilent QTOF 6546 mass spectrometer – QTOF MS (non-target analysis), both equipped with an Agilent Jet Stream electrospray ion source (Agilent Technologies, Santa Clara, CA, United States). The separation of analyses was performed in a 2.7 μm Poroshell 120 EC-C18 chromatographic column (3.0 × 100 mm, Agilent, Santa Clara, CA, United States). The mobile phase was composed of 0.5 mM ammonium fluoride in water and methanol. The gradient elution started with 5% methanol for 0.5 min and increased to 100% in 5 min. After 10 min at 100% methanol, the starting conditions were established and the column was equilibrated for 3 min prior to injection of the next sample. The flow rate was 0.6 ml/min and the column temperature was maintained at 40°C. 20 μl of sample extract was injected. For the target analysis, electrospray ionization (ESI) was operated in both positive and negative modes. Two specific ion transitions were monitored for each analyte in multiple-reaction monitoring mode (MRM): negative (Q1/Q3): apigenin 269/117; 205, chrysin 253/209; 135, daidzein 253/209; 135, naringenin 271/151; 119, morin 301/151; 125, quercetin 301/151; 179, positive: coumarin 147/103; 65, flavanone 225/121; 210, flavone 223/121; 178). The conditions for the electrospray were as follows: drying gas temperature 250°C, drying gas flow 8 l min**^–^**^1^, nebulizer pressure 35 psi, sheath gas temperature 400°C, sheath gas flow 12 l.min**^–^**^1^, capillary voltage ± 3500 V, nozzle voltage +0 V and −900 V. MassHunter Workstation software version 10.1 was used for data acquisition and post-run processing (Agilent, Santa Clara, CA, United States). For the non-target analysis, QTOF MS, operating in positive mode, was tuned using Swarm Autotune for the mass range *m/z* 50–750. Purine (m/z = 121.050873) was used as the reference molecule during the analysis to achieve the best mass accuracy. A single MS mode (ESI +, 5 spectra/s) in the range 50–600 m/z was chosen to perform metabolite profiling on the cell extracts. Agilent MassHunter Profinder version B.08.00 was used for recursive molecular feature extraction. The resulting features were evaluated with Agilent MassHunter Profiler Professional 15.1 (MPP) using the filter on the volcano plot algorithm with a cutoff of *p* < 0.05 and fold change > 1.5. Unique features revealed in the BPDO_AVS samples were exported to the inclusion list of an autoMS/MS method and acquired using two different collision energies (CE = 20 and 40 eV) and fragmentor voltage 140 V. The mass range was 50–600 m/z and MS/MS acquisition rate was 5 spectra/s.

### Sequence Deposition

Raw sequencing data were deposited in SRA under the BioProject no. PRJNA641877. Processed sequences were deposited in GenBank; these include 13 *bphA* SV: SV3 (GenBank accession no. MT671559), SV23 (MT671560), SV27 (MT671561), SV39 (MT671562), SV40 (MT671563, SV63 (MT671564), SV75 (MT671565), SV79 (MT671566), SV115 (MT671567), SV116 (MT671568), SV117 (MT671569), SV122 (MT671570), SV167 (MT671571), and the AVS region scaffold (MT680029).

## Results

### Diversity of *bphA* Genes in ^13^C-Enriched Metagenome After ^13^C-BP-SIP

**FIGURE 2:**
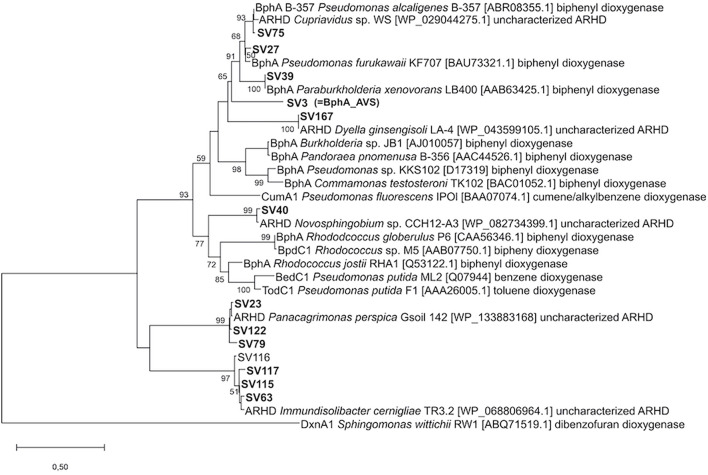
Phylogeny reconstruction of SVs of *bphA* amplicons in the context of typical ARHD large subunits. Accession numbers shown in brackets. The tree is drawn to scale, branch lengths represent the number of substitutions per site. Bootstrapping was used to test the tree topology (500 bootstraps), only bootstrap values > 50 are shown. The final dataset was comprised of 140 positions.

In total, 358 sequences spanning 13 unique *bphA* SVs were obtained by amplicon sequencing from the ^13^C-enriched metagenome after SIP with ^13^C-BP (further referred to as heavy DNA) (Supplementary Figure 2). SV3, the most abundant SV in the heavy DNA, accounted for 59% of all sequences and was also the most abundant in the total community DNA, where it accounted for 23% of sequences. The corresponding protein sequence was the only one in the data set with sequence identity <90% to hitherto described BphA sequences; in fact, it was only 77.4% identical to the closest detected sequence from *Cupriavidus* sp. WS and 76.7% to that of *Pseudomonas alcaliphila* B-367 (Vézina et al., 2008). Furthermore, in the phylogeny reconstruction based on the portion of ARHD large subunits corresponding to the amplicons sequenced, SV3 formed a separate branch outside the cluster formed by BPDOs from the strain *Par. xenovorans* LB400, *Ps. furukawaii* KF707, *Ps. alcaliphila* B-367, and the ARHD from *Cupriavidus* sp. WS (Figure 2). The other most abundant sequences in the heavy DNA were similar to the sequences of *Pseudomonas alcaliphila* JAB1 and *Ps. furukawaii* KF707 (designated SV27; 95.5% identity; accounted for 18% of all sequences) and *Panacagrimonas perspica* Gsoil 142 (designated SV23; 98.5% identity; accounted for 9% of all sequences). Other sequences accounted for <5% of all sequences and are depicted in Figure 2 and Supplementary Figure 2 in Supplementary Material.

SV3 was chosen for further investigation due to its low similarity to known BphA sequences and especially due to the unusual AA pattern of its region III, namely, Ala333, Val335, and Ser340 (Supplementary Figure 2). With this in mind, we designated the sequence *bphA_AVS*, and aimed to reveal the substrate specificity of the corresponding BPDO and thus predict its ecological function.

### Sequence Analysis of bphA_AVS and the Surrounding Region

**FIGURE 3:**
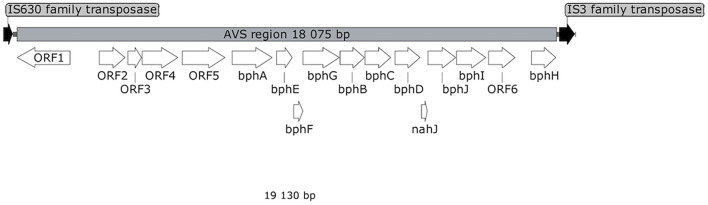
Map of AVS region. White arrows represent ORFs discussed in the text. ORFs encoding for putative transposases are shown in black.

Shotgun sequencing of the multiple-displacement-amplified ^13^C-metagenome yielded a 48.5 kbp scaffold which bore the *bphA_AVS* sequence. The DNA span of 18 kbp containing *bphA_AVS*, referred to hereinafter as the AVS region, is flanked on both ends by the remnants of IS3 family transposases, and contains 16 ORFs in total; its architecture is shown in Figure 3. The putative functions of the corresponding proteins inferred by homolog search analysis suggest their involvement in the metabolism of aromatic compounds as shown in Table 1. This region includes the *bphAE* genes encoding for a putative two-component Rieske-type BPDO, with the *bphA* gene corresponding to SV3. Putative ferredoxin and ferredoxin reductase genes (*bphF* and *bphG*, respectively) are located downstream of *bphAE*, followed by genes encoding for a *cis*-2,3-dihydrobiphenyl-2,3-diol dehydrogenase (*bphB*), biphenyl-2,3-diol 1,2-dioxygenase (*bphC*), and HOPDA-hydrolase (*bphD*). Putative products of ORFs *nahJ* (encoding for a putative 4-oxalocrotonate tautomerase), *bphH* (2-keto-4-pentenoate hydratase), *bphI* (4-hydroxy-2-oxovalerate aldolase), and *bphJ* [acetaldehyde dehydrogenase (acetylating)] are enzymes for the utilization of the catechol *meta*-cleavage intermediate, 2-hydroxymuconate (Sala-Trepat and Evans, 1971). Moreover, ORF1-ORF6 were identified within the AVS region, whose corresponding protein sequences show only a limited similarity to proteins characterized to date (in the range of 45–56%, Table 1). Except for the divergently oriented ORF1, which encodes a putative transcriptional regulatory protein, the deduced products of ORF2-ORF6 exhibit homology to enzymes involved in the metabolism of aromatic compounds, including cholesterol and 4-hydroxyphenyl acetate (Table 1).

**TABLE 1:** Functional assignment of deduced proteins encoded by ORFs within the AVS region.

**Gene designation**	**Product length [AA]**	**Closest UniProtKB/Swiss-Prot database hit (% of aa sequence similarity)**	**Presumed activity/function**	**Note**
ORF1	588	Transcriptional regulatory protein XylR (*Pseudomonas putida* TOL plasmid pWW0), P06519.1 (56%)	Transcriptional regulatory protein	Divergently oriented
ORF2	293	4,5:9,10-diseco-3-hydroxy-5,9,17-trioxoandrosta-1(10),2-diene-4-oate hydrolase HsaD (*Rhodococcus jostii* RHA1), Q9KWQ6.1 (45%)	*meta*-cleavage product hydrolase analogous to BphD in *bph*-encoded pathway	Unknown function – presumably degradation of an aromatic moiety
ORF3	157	NADH:flavin oxidoreductase 2, RutF (*Agrobacterium radiobacter* K84), B9JLT5.1 (55%)	NADH:flavin oxidoreductase	
ORF4	404	3-hydroxy-9,10-secoandrosta-1,3,5(10)-triene-9,17-dione 4-hydroxylase, oxygenase subunit HsaA (*Rhodococcus jostii* RHA1), Q0S811.1 (50%)	Hydroxylase of aromatic ring	
ORF5	486	4-hydroxyphenylacetate 3-monooxygenase oxygenase component HpaH (*Geobacillus* sp. PA-9), Q4L1M7.1 (53%)	Hydroxylase of aromatic ring	
*bphA*	458	Biphenyl 2,3-dioxygenase (*Pseudomonas furukawaii* KF707), Q52028.1 (87%)	Biphenyl 2,3-dioxygenase subunit α	Biphenyl upper degradation pathway: from biphenyl to benzoate
*bphE*	182	Biphenyl 2,3-dioxygenase subunit β (*Paraburkholderia xenovorans* LB400), P37334.3 (87%)	Biphenyl 2,3-dioxygenase subunit β	
*bphF*	109	Biphenyl 2,3-dioxygenase ferredoxin subunit (*Pseudomonas* sp. KKS102), Q52440.1 (79%)	Biphenyl 2,3-dioxygenase ferredoxin subunit	
*bphG*	413	Biphenyl 2,3-dioxygenase system ferredoxin-NAD(+) reductase component (*Paraburkholderia xenovorans* LB400), P37337.2 (79%)	Biphenyl 2,3-dioxygenase system ferredoxin-NAD(+) reductase	
*bphB*	279	*cis*-2,3-dihydrobiphenyl-2,3-diol dehydrogenase (*Comamonas testosteroni* B-356), 46381.1 (88%)	*cis*-2,3-dihydrobiphenyl-2,3-diol dehydrogenase	
*bphC*	293	Biphenyl-2,3-diol 1,2-dioxygenase (*Pseudomonas furukawaii* KF707), P08695.2 (81%)	Biphenyl-2,3-diol 1,2-dioxygenase	
*bphD*	286	2,6-dioxo-6-phenylhexa-3-enoate hydrolase (*Polaromonas naphthalenivorans* CJ2), A1VUV0.1 (79%)	2,6-dioxo-6-phenylhexa-3-enoate hydrolase	
*nahJ*	74	4-oxalocrotonate tautomerase (*Comamonas testosteroni* TA441), Q9RHM8.3 (72%)	4-oxalocrotonate tautomerase	Degradation of catechol *meta*-cleavage intermediate 2-hydroxymuconate yielding pyruvate and acetyl-CoA; missing 4-oxalocrotonate decarboxylase (e.g., NahK and its homologs DmpH, XylI, etc.)
*bphJ*	319	Acetaldehyde dehydrogenase (acetylating) 1 (*Azotobacter vinelandii* DJ, C1DMT1.1 (90%)	Acetaldehyde dehydrogenase (acetylating)	
*bphI*	335	4-hydroxy-2-oxovalerate aldolase 3 (*Dechloromonas aromatica* RCB), Q47B13.1 (92%)	4-hydroxy-2-oxovalerate aldolase	
ORF6	300	No significant hit in UniProtKB/Swiss-Prot database; in RefSeq database: non-characterized phenol degradation protein (*Paraburkholderia rhynchosiae*), WP_102636641.1 (80%); FdeA protein involved in flavonoid degradation (*Herbaspirillum seropedicae* SmR1), WP_041310253.1 (43%)	Unknown function	
*bphH*	278	2-keto-4-pentenoate hydratase 1 (*Dechloromonas aromatica* RCB), Q47HM0.1 (74%)	2-keto-4-pentenoate hydratase	

*The putative functions were assessed by blastp search against UniProtKB/Swiss-Prot (The UniProt Consortium, 2018) and RefSeq database (O’Leary et al., 2016), combined with the literature review.*

### Taxonomic Assignment of *bphA_AVS*-Host Organism

Based on a sequence search against the protein RefSeq database of all 59 detected ORFs localized in the *bphA_AVS*-bearing scaffold, the most likely genus assignment of the *bphA_AVS*-bearing organism is *Paraburkholderia*. Eighteen ORFs were found to have a protein identity higher than 90% to the reference proteins, of which 11 were assigned to the genus *Burkholderia*, 6 to *Paraburkholderia* and 1 to *Cupriavidus* according to the NCBI taxonomy system. All 11 hits for *Burkholderia* originated in the same genome of *Burkholderia* sp. OLGA172. Upon translating the NCBI taxonomy to GTDB taxonomy, which uses only genome information to classify prokaryotes, *Burkholderia* sp. OLGA172 was found to be reclassified into the *Paraburkholderia* genus, which made us conclude that the majority of proteins with high similarity to the references were of *Paraburkholderia* origin (Supplementary Table 1).

### BPDO_AVS-Mediated PCB Depletion

**FIGURE 4:**
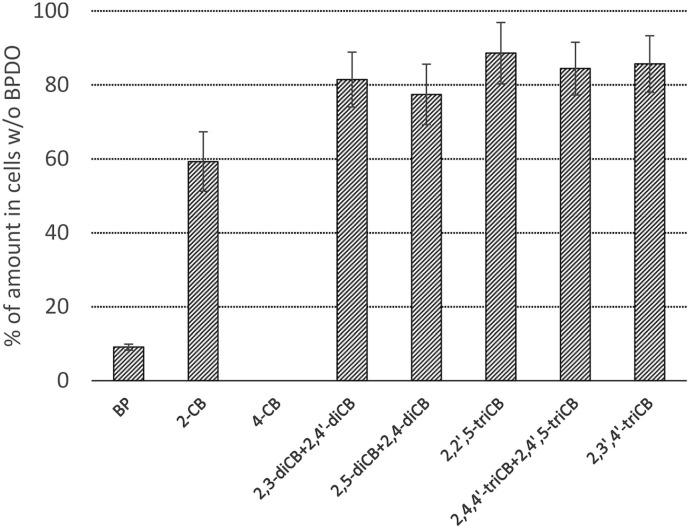
Depletion of PCBs by *E. coli* cells expressing BPDO_AVS. Only congeners with a statistically significant depletion are shown (*p* value < 0.05). The depletion rates were determined after the resting cell assay using IPTG-induced cells bearing both pQE31-bphAE_AVS and pYH31-bphFGBC_LB400 incubated with 0.0006% (w/v) Delor 103 for 24 h. The depletion rate of individual congeners is shown as the % of their amount in control microcosms bearing cells lacking BPDO_AVS. Error bars represent standard deviation (*n* = 4).

The PCB congeners contained in the Delor 103 mixture that were significantly (Student *t*-test, α = 0.05) depleted by BPDO_AVS-expressing *E. coli* cells compared to control cells lacking BPDO_AVS are displayed in Figure 4 and Supplementary Figure 4 in Supplementary Material. Among the congeners analyzed, the activity of BPDO_AVS led to the depletion of 2-CB, 4-CB, of the sum of 2,3-diCB + 2,4′-diCB, sum of 2,5-diCB + 2,4-diCB, 2,2′,5-triCB, 2,3′,4′-triCB, and the sum of 2,4,4′-triCB + 2,4′,5-triCB. The congeners whose depletion was not significant are listed in Supplementary Table 2.

### BPDO_AVS-Mediated SPM Transformation

**FIGURE 5:**
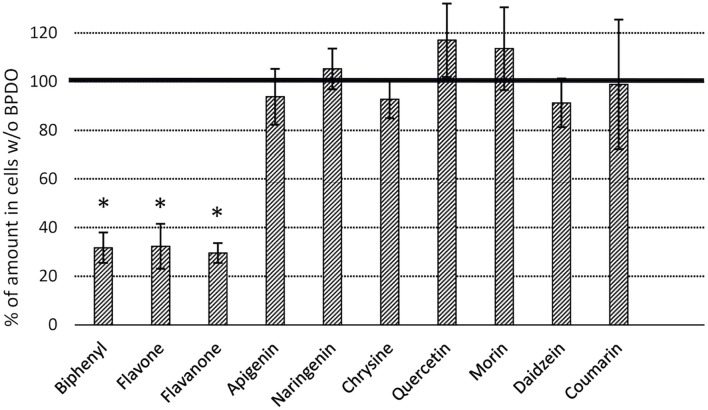
Depletion of flavonoids by *E. coli* cells expressing BPDO_AVS. The depletion rates were determined after the resting cell assay using IPTG-induced cells bearing both pQE31-bphAE_AVS and pYH31-bphFGBC_LB400 incubated with a 25 μM substrate for 24 h. The BPDO activity is shown as % of a SPM amount in control microcosms bearing cells lacking BPDO_AVS. (^∗^) statistically significant difference from the corresponding control (*p* value < 0.05). Error bars represent standard deviation (*n* = 4).

BPDO_AVS-bearing cells exhibited statistically significant depletions (% ± SD of SPM amount in control cell cultures lacking BPDO_AVS) of flavone (32 ± 9) and flavanone (30 ± 4), with percentage values being similar to those of BP (32 ± 6), as shown in Figure 5. Interestingly, when the substrate amounts of control cell cultures lacking BPDO_AVS were compared to the corresponding abiotic controls (no cells added), chrysin, apigenin, quercetin, and morin were found to be significantly depleted (by 17, 36, 53, and 43%, respectively), indicating substrate transformation mediated through enzymes other than BPDO_AVS.

**FIGURE 6:**
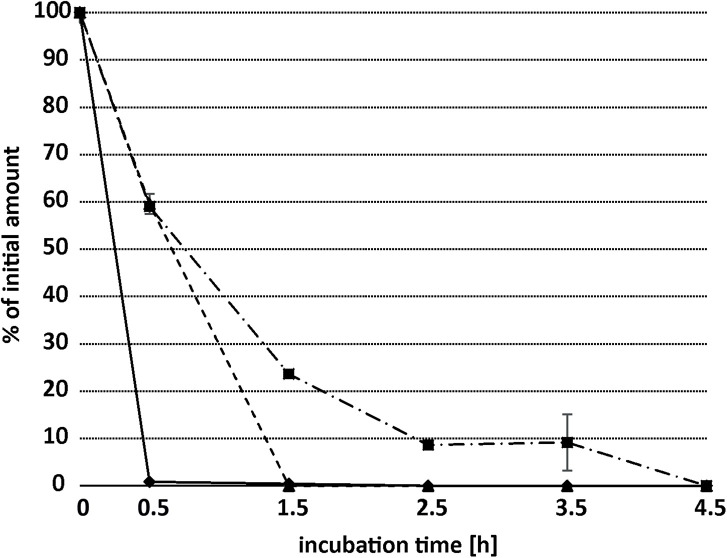
Time-dependent depletion of biphenyl (diamonds, solid line), flavone (triangles, dashed line), and flavanone (squares, dash-and-dot line) by the whole-cell extract of *E. coli* expressing BPDO_AVS. The depletion of substrates was determined using the crude extract of *E. coli* IPTG-induced cells bearing pQE31-bphAE_AVS and pYH31-bphFGBC_LB400, incubated with a 20 μM substrate. Error bars represent standard deviation (*n* = 3).

The depletion rates of biphenyl, flavone, and flavanone by BPDO_AVS were determined in crude whole cell extract of *E. coli* cells with active BPDO_AVS to avoid bias in the substrate availability for the BPDO holoenzyme in production cells caused by potential differences in the permeability of the bacterial cell wall for the substrates. As shown in Figure 6, 20 μM biphenyl was almost fully depleted (to 1% of the initial amount) after 0.5 h of incubation, with the calculated reaction rate of 33 pmol/min. Flavone and flavanone were fully depleted after 1.5 and 4.5 h of incubation with reaction rates for the initial 0.5 h of incubation of 13 and 14 pmol/min, respectively.

### Analysis of Transformation Products of Flavone and Flavanone

**FIGURE 7:**
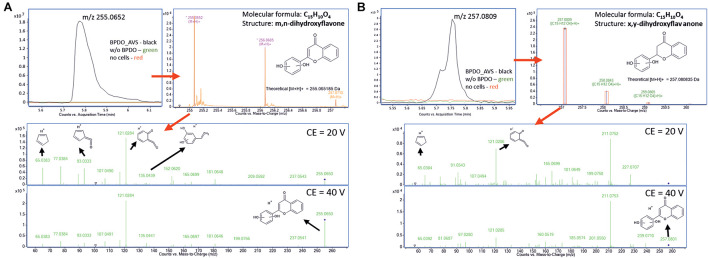
Analysis of products of flavone **(A)** and flavanone **(B)** transformation mediated by BPDO_AVS. Section of LC-MS chromatograms are shown of resting cell assay supernatants, together with the mass spectra of the corresponding compounds **(B)**, the fragmentation spectra of the major molecular features (m/z = 255.0652 and 257.0809, respectively) acquired using two different collision energies with suggested interpretation of MS/MS spectra and proposed structures of the corresponding compounds, determined as m,n-dihydroxyflavone and x,y-dihydroxyflavanone, respectively.

The high-resolution QTOF LC-MS differential analysis workflow was used to identify the products of the BPDO_AVS-mediated transformation of flavone and flavanone (Figure 7). The LC-MS analysis of flavone transformation products revealed a molecular feature with the *m*/*z* = 255.0652 found solely in the supernatant of the BPDO-bearing cells and not in either cells without BPDO or abiotic control. Based on the *m*/*z* and the isotopic pattern, the molecular formula of the corresponding compound was deduced as C_15_H_16_O_5_. Subsequent MS/MS fragmentation analyses suggest that the structure is m,n-dihydroxyflavone, which has the two hydroxyls on the B-ring (Figure 7). For flavanone, a molecular feature unique for BPDO-bearing cells with *m*/*z* = 257.0809 was found. The measured *m*/*z* and the isotopic pattern suggested the corresponding molecular formula as C_15_H_12_O_4_. Based on subsequent MS/MS fragmentation analysis, the compound was proposed as x,y-dihydroxyflavanone (Figure 7). Fragmentation spectra of the deduced compounds m,n-dihydroxyflavone and x,y-dihydroxyflavanone showed the same pattern as it was reported previously for B-ring-hydroxylated flavones/flavanones (Shimada et al., 2021). Therefore, we assume that BPDO_AVS mediated the hydroxylation of the B-ring of flavone and flavanone; nevertheless, the precise position of the two hydroxyls could not be determined due to the inaccessibility of commercial analytical standards, hence labeling m,n and x,y is used herein.

## Discussion

Recalcitrant organic compounds are not a negligible portion of organic carbon in soils, be it plant-derived organic compounds in vegetated soils (Cortes-Tolalpa et al., 2018; Hu et al., 2018), or persistent organic pollutants in contaminated soils (Mackova et al., 2006). Our understanding of the biodegradation of recalcitrant compounds requires a complex insight into the indigenous soil microbial populations, their enzymatic equipment, and specific functions that the enzymes perform. Molecular techniques such as SIP in tandem with metagenomics not only permit microbial ecologists to assign a specific function to individual, metabolically active microbial taxa, but also to explore the function of specific genes (Uhlik et al., 2013; Hernández et al., 2017). In this research, we have followed up on our previous study, in which we used SIP to identify metabolically active BP-utilizing bacteria in the legacy PCB-contaminated soil (Uhlik et al., 2012), and analyzed the prevailing *bphA* sequence in the heavy DNA. Unlike the other SVs in the data set, whose protein sequences were similar to previously studied BphAs from pure cultures, the prevailing SV3 sequence was uncommon in its deduced primary structure. In addition to the relatively low sequence similarity of the translated SV3 sequence to known BphAs (Table 1), the AA pattern in region III differed as well (Supplementary Figure 2). Based on multiple previous reports of Sylvestre et al. (Mondello et al., 1997; Barriault et al., 2002; Barriault and Sylvestre, 2004; Vézina et al., 2008; Mohammadi et al., 2011), this region is crucial for the substrate specificity of the BPDO. More recent reports also showed that diverse BPDOs play different ecological roles associated with the transformation of various types of recalcitrant compounds, such as flavonoids, in addition to PCBs (Pham and Sylvestre, 2013). With this in mind, the complete *bphA* coding sequence corresponding to SV3 and the surrounding region were retrieved from the metagenome and the substrate specificity of the corresponding BPDO_AVS toward PCBs and flavonoids was determined.

The AVS region (Figure 3) is flanked on both ends by the remnants of IS3 family transposases, therefore it presumably arose from horizontal gene transfer. ORF4 and ORF2 exhibit a certain level of sequence homology with the enzymes involved in steroid degradation found in *R. jostii* RHA1 and other actinobacteria, specifically the oxygenase subunit HsaA, which hydroxylates aromatic ring A in the cholesterol-degradation intermediate (50% similarity to ORF4), and the meta-cleavage product hydrolase HsaD (45% similarity to ORF2) (Van der Geize et al., 2007). Nevertheless, no ORF potentially encoding for a functional homolog of HsaC, enabling the *meta*-cleavage of the product of the HsaA-mediated reaction, has been found in the AVS region. The following ORF5 encodes for another putative monooxygenase hydroxylating an aromatic ring.

The predicted protein sequences of the seven subsequent ORFs *bphAEFGBCD* resemble the enzymes of the BP upper-degradation pathway of known BP/PCB degraders such as *Paraburkholderia xenovorans* LB400 (Erickson and Mondello, 1992), *Pseudomonas furukawaii* KF707 (Taira et al., 1992), *Ps. alcaliphila* JAB1 (Ridl et al., 2018), *Acidovorax* sp. KKS102 (Fukuda et al., 1994), *Rhodococcus jostii* RHA1 (Masai et al., 1995), *Rhodococcus* sp. strain R04 (Yang et al., 2007), etc. In bacteria, this pathway enables the activation and further degradation of the otherwise recalcitrant BP aromatic structure by dihydroxylation, extradiol cleavage, and hydrolysis of the corresponding intermediate, while yielding benzoic acid and 2-hydroxy-penta-2,4-dienoic acid. In the phylogeny reconstruction, the full-length BP 2,3-dioxygenase large subunit encoded by the *bphA_AVS* gene shared a common ancestor with a discrete cluster formed by BphA sequences from the strains *Ps. furukawaii* KF707, *Acinetobacter* sp. KKS102, *Ps. alcaliphila* JAB1, *Pandoraea pnomenusa* B-356, *Par. xenovorans* LB400, and *Cupriavidus oxalaticus* A5 (Supplementary Figure 3). These were separated from a cluster formed by BphA of *R. jostii* RHA1 (Masai et al., 1995) and *Rhodococcus* sp. M5 (Wang et al., 1995), as well as by BphAs from both *Rhodococcus erythropolis* TA431 (Chung et al., 1994) and *Rhodococcus* sp. R04 (Yang et al., 2007). Subsequent ORFs *nahJ*, *bphH*, *bphI*, and *bphJ* encode enzymes enabling utilization of the catechol *meta*-cleavage pathway intermediate 2-hydroxymuconate (Austen and Dunn, 1977). Nevertheless, due to the absence of a 2-oxo-3-hexenedioate decarboxylase homolog in the AVS region, the corresponding pathway would not be complete *per se* and would require complementation *in trans*. The protein product of ORF6 might be the missing step to potentially complement the missing function, however, such an assumption is highly hypothetical with no experimental evidence.

Taking into account the entire *bphA_AVS*-bearing scaffold, the *bphA_AVS*-bearing organism is very likely a bacterium of the genus *Paraburkholderia*, based on the high protein identity of ORFs to those of *Paraburkholderia* (Supplementary Table 1). *Paraburkholderia* identity is further supported by a high prevalence of *Paraburkholderia* (formerly *Burkholderia*) 16S rRNA gene sequences in this heavy DNA, as was reported previously (Uhlik et al., 2012).

To investigate the substrate preferences of the metagenome-extracted BPDO_AVS, the gene cluster *bphAE_AVS* was heterologously co-expressed with the gene cluster *bphFGBC* from *Par. xenovorans* LB400 (Chebrou et al., 1999; Vézina et al., 2007). Such a whole-cell-based approach to infer the catalytic activity of various BPDOs has been already successfully applied (Chebrou et al., 1999; Shindo et al., 2003; Barriault et al., 2004; Kagami et al., 2008; Pham et al., 2012; Toussaint et al., 2012). The advantage of BPDO co-expression with the genes BphB and BphC is the possibility to easily verify the presence of the active BPDO enzyme pathway through the evolution of the yellow BP transformation product HOPDA (Kumamaru et al., 1998; Barriault and Sylvestre, 2004).

As is evident from the depletion assay performed with the PCB mixture Delor 103, BPDO_AVS was able to catalyze transformation of only several lower chlorinated congeners (Figure 4). Predictably, BPDO_AVS exhibited a strong preference for 4-CB, resulting in its complete depletion under the tested conditions; 4-CB is a PCB congener considered to be normally transformed at rates similar to or higher than BP by most BPDOs (Kumamaru et al., 1998). All other depleted congeners had a chlorine substitution in the *ortho* and/or *para* positions. Such a finding of relatively narrow regioselectivity is in strong contrast with the phylogenetically close relatives BPDO_B-356 (Gómez-Gil et al., 2007), BPDO_LB400 (Mondello, 1989) and BPDO_JAB1 (Vergani et al., 2019; Garrido-Sanz et al., 2020).

**FIGURE 8:**
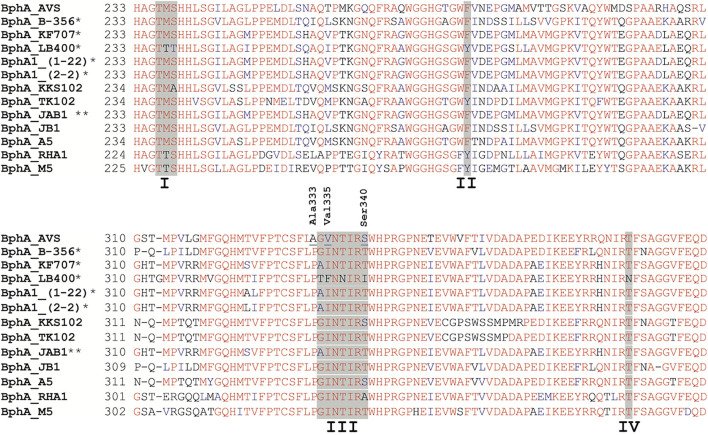
Multiple sequence alignment of functionally characterized BphA protein sequences closely related to BphA_AVS. Regions I, II, III, and IV as discussed by Mondello et al. (1997) and Vézina et al. (2008) are highlighted with a gray background. The BphA_AVS residues Ala333 and Val335 discussed in the text are marked. Highly conserved regions are shown in red. Residues with greater than 50% identity are shown in blue. (*) Transformation of flavonoids by BPDO reported in the literature, (**) transformation of flavone and flavanone proven (Zubrova et al., 2021).

However, BphA_AVS bears an unusual AA motif in region III, as shown in Figure 8, which was the rationale for choosing *bphA_AVS* as the target of this study for determining its substrate specificity. To the best of our knowledge, the BphA_AVS Ala333 preceding region III is a residue totally unique in BphA sequences, in which proline is most commonly present (Vézina et al., 2008; Figure 8). Another uncommon AA residue in BphA_AVS is Val335, which has only been found in a few ARHD sequences other than BPDO, e.g., benzene 1,2-dioxygenase large subunit BedC1 (Tan et al., 1993) and in a couple of environmental sequences (Vézina et al., 2008). The Ser340 in the BphA_AVS sequence is found commonly in BPDO sequences nevertheless, its combination with Ala333 and Val335 is unique. Pham et al. (2012) linked the presence of Gly333 and Ile334 in region III of BphA_B-356, corresponding to Thr335 and Phe336 in BphA_LB400 (Figure 8), to the superior ability of the BPDO_B-356 to transform larger substrates, such as DDT, 2,6-di-*tert*-butylphenol, and flavonoids. The Phe336 found in BphA_LB400 hinders the productive binding of bulkier substrates due to the larger side chain compared to Ile334 of BphA_B-356, and, simultaneously, the substitution of Thr335 of BphA_LB400 for Gly333 in BphA_B-356 results in higher protein structural flexibility during substrate binding (Pham et al., 2012). Gly334 and Val335 at the corresponding positions of BphA_AVS, the latter being even less bulky than Ile334 in BphA_B-356 and also totally unique in BphA sequences, led us to hypothesize that BphA_AVS could accommodate more bulky substrates into the binding pocket, including the chromone/chromanone moiety of flavonoids. To test this hypothesis, we proceeded to investigate the substrate preference of the BPDO_AVS toward flavonoids.

Our data demonstrate that the BPDO_AVS was capable of efficiently transforming flavone and flavanone (Figure 5). The initial reaction rates of both flavone and flavanone were approx. 40% of that determined for BP (Figure 6). No transformation was observed for daidzein (a representative of isoflavonoids), naringenin (a flavanone), chrysin and apigenin (hydroxylated flavones), quercetin and morin (flavonols), and coumarin (structure analogous to the chromene moiety of flavones). The transformation of flavonoids in control *E. coli* cells cultures lacking BphA_AVS, which could not be attributed to the activity of BPDO_AVS, could be explained by the action of the enzymes encoded by pYH31-bphFGBC_LB400. For instance, extradiol dioxygenases BphC from *Rhodococcus* sp. R04, *Dyella ginsengisoli* LA-4, and *Comamonas* sp. SMN4 were found to transform substituted catechols in addition to 2,3-dihydroxybiphenyl (Yang et al., 2008; Li et al., 2009; Lee and Kwon, 2016). Therefore, BphC_LB400 could possibly also act on the catechol moieties present in quercetin. Alternatively, the non-BPDO-mediated depletion of flavonoids could also be caused by other non-specific enzymes indigenous to the *E. coli* genetic background. For instance, the extradiol dioxygenases MhpB and HcaB were reported to possess relatively broad substrate specificities (Spence et al., 1996; Díaz et al., 1998), and could act on the hydroxylated aromatic moieties within the structure of the flavonoids discussed here.

The products of both flavone and flavanone transformation revealed here suggest that BPDO_AVS mediated the formation of dihydrodiol on the B-ring. In general, the *ortho-meta* dihydroxylation of an aromatic ring by BPDOs results in the production of *cis*-dihydrodiols that are further converted to the respective aromatic diols *via* the action of BphB (Barriault et al., 1999; Li et al., 2009). Analogously, we assume that BphB encoded on the plasmid pYH31-bphFGBC_LB400 mediated such transformation of flavone- and flavanone-dihydroxylated intermediates produced by BPDO_AVS, resulting in the re-aromatization of the dihydrodiol B-ring, similarly as in the studies of Seeger et al. (2003) and Kagami et al. (2008). Flavone and flavanone, unlike the rest of the flavonoids tested, lack hydroxyl groups on the B-ring. Therefore, the absence of substituents at the B-ring seems to be determinative for the activity of BPDO_AVS. On the other hand, saturation of the C2 and C3 bond, which differentiates flavanone from flavone, does not seem to influence the selectivity of the BPDO_AVS enzyme. Attack of the B-ring in the flavonoid structure by Rieske-type ARHD has also been reported for other BPDOs closely related to BPDO_AVS (Supplementary Figure 3). For instance, the BPDO from *Ps. furukawaii* KF707 was demonstrated to catalyze the dihydroxylation of the flavone B-ring at positions C2′ and C3′ (Kim et al., 2003). Interestingly, the study of Han et al. (2005) reported that with flavanone, 6-hydroxyflavanone and 7-hydroxyflavanon, BPDO_KF707 acted as a monooxygenase introducing an epoxide functional group between C2′ and C3′ of the B-ring. The attack on the B-ring of flavone and flavanone, followed by its subsequent transformation yielding 4-oxo-4H-chromene-2-carboxylic acid, was also reported in our recent study with the PCB-degrader *Ps. alcaliphila* JAB1 which has a KF707-type BPDO (Zubrova et al., 2021). Similarly, the formation of *cis*-diols on the B-ring of flavone, flavanone, and isoflavone, was reported for the BPDO from *Pan. pnomenusa* B-356, with k_cat_ and k_cat_/K_m_ values comparable to those of BP (flavone and isoflavone) or even higher (flavanone). In contrast, BPDO_LB400 was only poorly active toward all of these simple flavonoids (Pham et al., 2012). Apart from wild-type enzymes, several artificially engineered BPDOs have been reported with improved substrate preferences toward flavonoids. Of those, a BphA_LB400 mutant BphA_*p*4 is worth mentioning, which was prepared by the substitution of Thr335 and Phe336 in region III for less bulky Ala and Met, respectively, that efficiently transformed isoflavone through the dihydroxylation of the B-ring, and flavone and flavanone through the dihydroxylation of both B- and C-rings (Pham et al., 2012). Similarly, BphA_KF707-derivatives BphA1 (1–22) and BphA1 (2-2) mediated dihydroxylation at positions C1′ and C2′ of the B-ring of both 7-hydroxyflavone and chrysin (Kagami et al., 2008). Another BphA_KF707-derivative BphA1 (2072) was shown to transform flavone, flavanone, 6-hydroxyflavone, 6-hydroxyflavanone, 7-hydroxyflavone, and chalcone also through dihydroxylation of the B-ring (Misawa et al., 2002; Shindo et al., 2003).

In contrast, naphthalene dioxygenase NdoB from *Pseudomonas* sp. strain NCIB 9816-4 was demonstrated to hydroxylate the A-ring of flavone and isoflavone (Seo et al., 2010). As shown in the phylogeny reconstruction of full-length ARHD large subunits in Supplementary Figure 3 in Supplementary Material, NdoB is distant from the cluster formed by model BPDO large subunits, including BphA_AVS.

Several studies also aimed to link BPDOs with the bacterial transformation of SPMs at the level of regulating gene/protein expression. For instance, in the survey performed with a diverse spectrum of aromatic substances including the flavonoids myricetin and naringenin, none of the tested flavonoids were able to significantly increase the induction of the *bphA* gene in either *Pseudomonas* sp. Cam-1 or *Par. xenovorans* LB400 (Master and Mohn, 2001), and of other SPMs tested, only salicylic acid induced *bphA* in the Cam-1 strain. In contrast, Pham et al. (2015) found a wide spectrum of flavonoids to be efficient promoters of the BPDO activity of the rhizosphere bacterium *Rhodococcus erythropolis* U23A. Flavone, isoflavone, and flavanone were even better inducers of the BP catabolic enzymes than BP itself. Moreover, the PCB-degrading activity in *R. erythropolis* U23A was significantly induced by *Arabidopsis thaliana* root exudates in hydroponics-based experiments, which was attributed to flavanone present in the exudates (Toussaint et al., 2012). In our aforementioned study with *Ps. alcaliphila* JAB1, the BPDO borne by this strain was induced by multiple SPMs including flavonoids, umbelliferone (a coumarin), monoterpenes, and phenolic acids (Zubrova et al., 2021).

The poor catalytic efficiency of BPDO_AVS toward PCBs congeners on the one hand and the ability to efficiently transform flavonoids on the other are further evidence of the role of BPDOs in the transformation of specific SPMs. The demonstrated ability of BPDO_AVS to mediate dihydroxylation of flavonoids supports the hypothesis primed by Focht (1995) and further discussed by Pham et al. (2012) of the divergent evolution of individual BPDOs to serve different functions in the environment. These functions can include, apart from the degradation of recalcitrant pollutants such as PCBs, an involvement of soil BPDOs in environmental processes that are mediated by flavonoids (Shaw et al., 2006; Cesco et al., 2012). Presumably, these could be (i) the ability to utilize a pool of soil flavonoids as growth substrates, (ii) the transformation of those flavonoids which are potentially inhibitory for the host microorganisms, (iii) the modulation of flavonoid-mediated signaling processes in soil during the formation of nodules with nitrogen-fixing rhizobacteria, mycorrhiza, interactions with phytopathogens, and others (Shaw et al., 2006; Subramanian et al., 2007; Cesco et al., 2012; Liu and Murray, 2016). Given rather limited capacity of the BPDO_AVS to transform PCBs and the genetic context of the whole AVS region, especially the presence of ORF2-6 with the putative function in degradation of an aromatic moiety, we hypothesize that the utilization of biphenyl by BPDO_AVS is a result of exaptation of the enzyme. As for the utilization of flavonoids as a sole carbon source, there is a lack of direct experimental evidence that BPDOs are a determinative factor for such an environmental function. To the best of our knowledge, the only genetic determinant of flavonoid utilization reported to date is the *fde* locus from the diazotrophic endophyte *Herbaspirillum seropediacae* SmR1 (Marin et al., 2013). This bacterium is capable of utilizing the flavanone naringenin through monohydroxylation on the C8 of the A-ring followed by A-ring fission and further transformation (Marin et al., 2016). The initial hydroxylation was shown to be catalyzed by the complex FdeED comprising putative FAD-binding monooxygenase (FdeE) and the non-heme Rieske-protein electron carrier FdeD. Hypothetically, in the AVS-bearing organism, the putative products of ORF2-ORF6 could complement such activation of the A-ring *via* hydroxylation and subsequent fission, thus enabling the degradation of the rest of the flavonoid backbone. Such a hypothetic functional analogy between the *fde* pathway in *H. seropediacae* and the AVS region raises the need for further investigation. In particular, potential involvement of the putative proteins ORF2-ORF6 borne by the AVS region in the degradation of flavonoids should be studied, together with the regulation of the expression of these genes in response to SPMs.

## Conclusion

Based on the genetic context and the enzymatic activity of BPDO_AVS toward flavonoids demonstrated here, we assume that BPDO_AVS primarily evolved for the transformation of flavonoids present in the soil/rhizosphere of various characters, either released, for instance, as part of the root exudates (Cesco et al., 2010) or originating from the plant necromass (Kraus et al., 2003; Iwashina, 2015). The putative protein products of ORF2-ORF5 in tandem with those encoded downstream of *bphAEFG* could be involved in the further transformation of the resulting flavonoid moiety, which remains to be confirmed.

## Data Availability Statement

The datasets presented in this study can be found in online repositories. The names of the repository/repositories and accession number(s) can be found in the article/Supplementary Material.

## Author Contributions

JSu and OU designed the experiments and wrote the manuscript. JSu, AZ, JC, JW, KM, MH, JSe, and KS conducted the experiments and generated the data. JSu and MS analyzed the data. TC and OU provided the equipment and supplies. All authors contributed to the article and approved the submitted version.

## Conflict of Interest

The authors declare that the research was conducted in the absence of any commercial or financial relationships that could be construed as a potential conflict of interest.

## Publisher’s Note

All claims expressed in this article are solely those of the authors and do not necessarily represent those of their affiliated organizations, or those of the publisher, the editors and the reviewers. Any product that may be evaluated in this article, or claim that may be made by its manufacturer, is not guaranteed or endorsed by the publisher.

## Footnotes

1
https://github.com/indraniel/sff2fastq

2
https://sourceforge.net/projects/bbmap/

## Abbreviations

AA: amino acid
ARHD: aromatic ring-hydroxylating dioxygenase
HOPDA: 2-hydroxy-6-oxo-6-phenyl-2,4-hexadienoic acid
BP: biphenyl
BPDO: biphenyl 2,3-dioxygenase
CB: monochlorobiphenyl
diCD: dichlorobiphenyl
triCB: trichlorobiphenyl
IPTG: isopropyl- β -D-1-thiogalactopyranoside
PCB: polychlorinated biphenyl
SIP: stable isotope probing
SV: sequence variant.
