# Supplementary material for: Predominant Biphenyl Dioxygenase From Legacy Polychlorinated Biphenyl (PCB)-Contaminated Soil Is a Part of Unusual Gene Cluster and Transforms Flavone and Flavanone
Source: Front Microbiol. 2021 Oct 14;12:644708. doi: 10.3389/fmicb.2021.644708 (PMC8552027; doi:10.3389/fmicb.2021.644708)
Supplement: Supplementary file 1 [file Data_Sheet_1.DOCX]

**Supplementary material**

**Predominant biphenyl dioxygenase from legacy polychlorinated biphenyl (PCB)-contaminated soil is a part of unusual gene cluster and transforms flavone and flavanone**

Jachym Suman^1,🖂^, Michal Strejcek^1^, Andrea Zubrova^1^, Jan Capek^1^, Jiri Wald^1^, Klara Michalikova^2^, Miluse Hradilova^3^, Kamila Sredlova^2^, Jaroslav Semerad^2,4^, Tomas Cajthaml^2,4^, Ondrej Uhlik^1,🖂^

^1^*University of Chemistry and Technology, Prague, Faculty of Food and Biochemical Technology, Department of Biochemistry and Microbiology, Prague, Czech Republic*

^2^*Institute of Microbiology, Academy of Sciences of the Czech Republic, v.v.i., Prague, Czech Republic*

^3^*Institute of Molecular Genetics, Academy of Sciences of the Czech Republic, v.v.i., Prague, Czech Republic*

*^4^Institute for Environmental Studies, Faculty of Science, Charles University, Prague, Czech* Republic

| 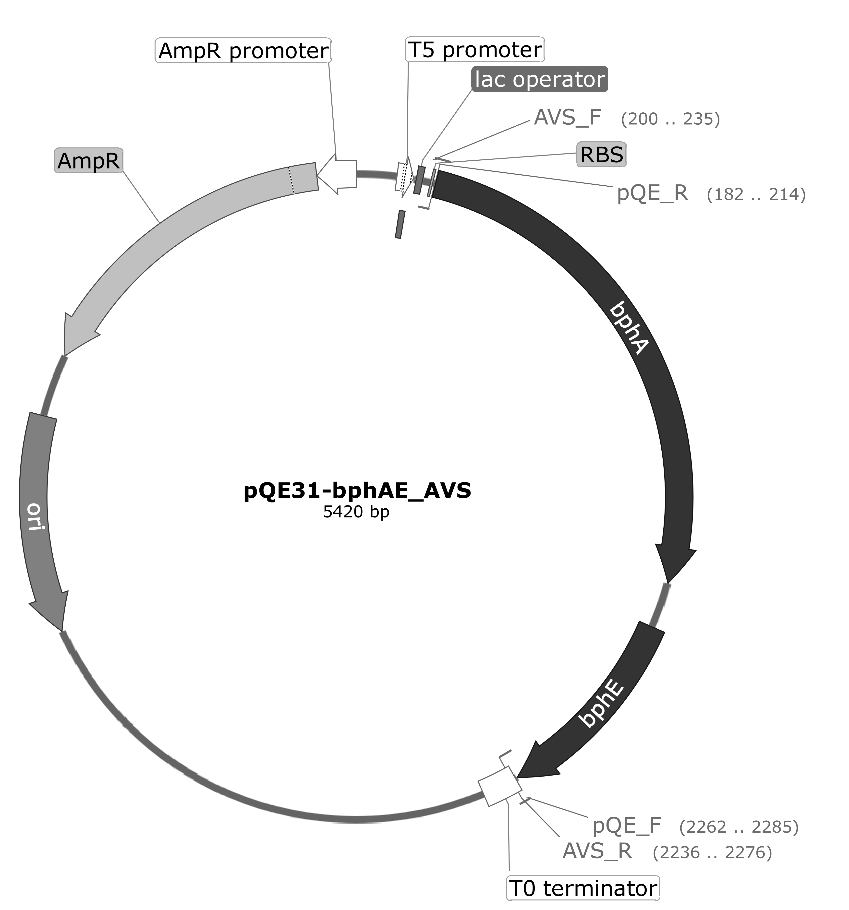 |
| --- |
| *Figure S1: The map of the plasmid pQE31-bphAE_AVS used for the heterologous expression of the bphAE_AVS genes in* E. coli. The plasmid enabled IPTG-inducible selection of transgenes and the ampicillin-based selection of transformants. The primers used for the construction as described in the Methods section are indicted. AmpR, β-lactamase gene; RBS, *E. coli* ribosome binding site; ori, origin of replication. |

| SV3(59%) 1 QFCSDMYHAGTMSHLSGILAGLPPELDLSNAQTPMKGQQFRAQWGGHGTGWFVNE--PGMAMVTTGSKVAQYWMDSPAARHAQSRLGST-MPVLGMFGQHMTVFPTCSFLAGVNTIRSWHPRGPNETEVWVFTIV  SV167(<1%) 1 QFCSDMYHAGTMSHLSGILAGMPPDMDLSQAPMPTAGYQFRAAWGGHGTGWFEDE--AGFLATVVGPKAAAYWYGSEASKRTAERLADRLPRFLRMSGQHMTVFPTCSFLPGINTIRSWHPRGPNEIEVWAFTVV  ARHD_LA-4 231 QFCSDMYHAGTMSHLSGILAGMPPDMDLSQAPMPTAGYQFRAAWGGHGTGWFEDE--AGFLATVVGPKAAAYWYGSEASKRTAERLADRLPRFLRMSGQHMTVFPTCSFLPGINTIRSWHPRGPNEIEVWAFTVV |
| --- |
| SV27 (18%) 1 QFCSDMYHAGTMSHLSGILAGMPPEMDLSDAQVPTKGNQFRASWGGHGTGWYVDE--PGMLMAVMGPKVTQYWTEGPAADLAEKRLGHT-MPARRMFGQHMSVFPTCSFLPAINTIRTWHPRGPNEIEVWAFTLV SV75 (3%) 1 QFCSDMYHAGTMSHLSGILAGMPPEMDLSNAQVPTKGNQFRANWGGHGTGWFVDE--PGMLMAVMGPKVTQYWTEGPAADLAERRLGQT-MPVRRMFGQHMNIFPTCSFLPAINTIRSWHPRGPNEIEVWAFTLV  BphA B-357 104 QFCSDMYHAGTMSHLSGILAGMPPEMDLSNAQVPTKGNQFRANWGGHGTGWFVDE--PGMLMAVMGPKVTQYWTEGPAADLAEKRLGQT-MPVRRMFGQHMNIFPTCSFLPAINTIRSWHPRGPNEIEVWAFTLV  ARHD WS 226 QFCSDMYHAGTMSHLSGILAGMPPEMDLSNAQVPTKGNQFRANWGGHGTGWFVDE--PGLLMATMGPKVTQYWTEGPAADLAEKRLGQT-MPVRRMFGQHMNIFPTCSFLPAVNTIRSWHPRGPNEIEVWAFTLV  BphA KF707 226 QFCSDMYHAGTMSHLSGILAGMPPEMDLSHAQVPTKGNQFRAGWGGHGSGWFVDE--PGMLMAVMGPKVTQYWTEGPAADLAEQRLGHT-MPVRRMFGQHMSVFPTCSFLPAINTIRTWHPRGPNEIEVWAFTLV  SV39 (1%) 1 QFCSDMYHAGTTTHLSGILAGIPPEMDLSQAQIPTKGNQFRAAWGGHGSGWYVDE--PGSLLAVMGPKVTQYWTEGPAAELAEQRLGHTGMPVRRMVGQHMTIFPTCSFLPTFNNIRIWHPRGPNEIEVWAFTLV  BphA_LB400 226 QFCSDMYHAGTTTHLSGILAGIPPEMDLSQAQIPTKGNQFRAAWGGHGSGWYVDE—-PGSLLAVMGPKVTQYWTEGPAAELAEQRLGHTGMPVRRMVGQHMTIFPTCSFLPTFNNIRIWHPRGPNEIEVWAFTLV |
| SV23 (9%) 1 QFCSDMYHA-PLSHMSAILAVLPDGVPPEAARWPTEGLQWRSPNAGHGTGFHTPDDQGQLLAAIVGPSVAQYLME-SRP-RVAARLGNA--RTTAVNGAHMTIFPTCSFLPGINTLRVWHPRGPNEIEVWAMAIV SV79 (4%) 1 QFCSDMYHA-PLSHMSAILAVLPEGVPPEAAQWPTEGLQWRSPNAGHGAGWHTPDDQGQLLGAIVGPSVAQYLME-SRP-RVTARLGNE--RTTAVNGAHMTIFPTCSFLPGINTLRVWHPRGPNEIEVWAMAIV SV122 (2%) 1 QFCSDMYHA-PLSHMSAILAVLPDGVPPEAAQWPTEGLQWRSPNAGHGTGFHTPDDQGQLLGAIVGPSVAQYLME-SRP-RVAARLGNA--RTTAVNGAHMTIFPTCSFLPGINTLRVWHPRGPNEIEVWALAIV ARHD Gsoil 142 212 QFCSDMYHA-PLSHMSAILAVLPEGVPPEAAQWPTEGLQWRSPNAGHGTGFHTPDDQGQLLAAIVGPSVAQYLME-SRP-RVAARLGNA--RTTAVNGAHMTIFPTCSFLPGINTLRVWHPRGPNEIEVWAMAIV |
| SV40 (<1%) 1 QFCSDMYHAGTVSHLSGILAGLPEDVELGDVQIPTSGFQFRSDGGGHGTGFYVAE--PAMLQAIMGPRVTEYWTDSPAAQTAESRLGSAE-RARELMAQHMTIFPTCSFLPGINTVRTWHPRGPGEIEVWAFTVV  ARHD CCH12-A3 213 QFCSDMYHAGTVSHLSGILAGLPEDVELGDVQIPTSGFQFRSDGGGHGTGFYVAE--PAMLQAIMGPRVTEYWTDSPAAQAAESRLGSAE-RACKLMAQHMTIFPTCSFLPGINTVRTWHPRGPGEIEVWAFTVV  BphA JB1 226 QFCSDMYHAGTMSHLSGVLAGLPPEMDLTQIQLSKNGNQFRSAWGGHGAGWFIND--SSILLSVMGPKITQYWTQGPAAEKAAS-VPQL--PILDMFGQHMTVFPTCSFLPGINTIRTWHPRGPNEVEVWAFVLV  BphA B-356 226 QFCSDMYHAGTMSHLSGVLAGLPPEMDLTQIQLSKNGNQFRSAWGGHGAGWFIND--SSILLSVVGPKITQYWTQGPAAEKAARRVPQL--PILDMFGQHMTVFPTCSFLPGINTIRTWHPRGPNEVEVWAFVLV  BphA KKS102 227 QFCSDMYHAGTMAHLSGVLSSLPPEMDLTQVQMSKNGSQFRAAWGGHGSGWFIND--AAILMAVMGPKITQYWTQGPAAEKAAKRLNQM--PTQTMFGQHMTVFPTCSFLPGINTIRSWHPRGPNEVECGPSWSS  BphA TK102 227 QFCSDMYHAGTMSHVSGVLASLPPNMELTDVQMPKNGAQFRAAWGGHGTGWYIND--PDILMAVMGPKITQFWTEGPAAEKAAKRLNQM--PTQTMFGQHMTVFPTCSFLPGINTIRTWHPRGPNEVECGPSWSS |
| SV63 (1%) 1 QFCSDMYHA-PFSHSSPVLASLPPDVDPGQAGWPMKGKQFSS-ALGHGTGFFMDT--PEFLFPLLGETAAGYYMQ-SGATAVTERLGDV--RGVRMNGAHMTVFPTLSFLPGINTLRVWHPRGPGEIEIWAWTLV SV115 (<1%) 1 QFCSDMYHA-PFSHSSPVLASLPPDIDPSQAGWPMKGKQFSS-ALGHGTGFFMDT--PEFLFPLLGETAAGYYMQ-SGATAVTERLGDV--RGVRMNGAHMTVFPTLSFLPGINTLRVWHPRGPDEIEIWAWTLV SV117 (<1%) 1 QFCSDMYHA-PFSHSSPVLASLPPDIDPSQAGWPMKGKQFSS-ALGHGTGFFMDT--PQFLFPLLGETAAGYYLQ-SGATAVTERLGDV--RAQRMNGAHMTVFPTLSFLPGINTLRVWHPRGPDEIEIWAWTLV SV116 (<1%) 1 QFCSDMYHA-PFSHSSPVLASLPPDIDPSQAGWPMKGKQFSS-ALGHGTGFFMDT--PEFLFPLLGETAAGYYMQ-SGA-AAAERLGEV--RGARMNGAHMTVFPTLSFLPGINTLRVWHPRGPDEIEIWAWTLV  ARHD TR3.2 205 QFCSDMYHA-PFSHSSPVLASLPPDIDPGQAGWPMKGKQFSS-ALGHGTGFFMDT--PEFLFPLLGETAAGYYMQ-SGATAVTERLGEL--RGVRMNGAHMTVFPTLSFLPGINTLRVWHPRGPDEIEIWAWTLV  BphA P6 218 QFCSDMYHVGTTSHLSGLLAGLPDEIDIREVQPPTTGIQYSAPWGGHGSGFYIGE--MGTLAAVMGMKILEYYTSGPAAEKAAKRLGSAV-RGSQATGQHMTVFPTCSFLPGINTIRTWHPRGPHEIEVWSFTVV |
| BphA RHA1 217 QFCSDMYHAGTTSHLSGILAGLPDGVDLSELAPPTEGIQYRATWGGHGSGFYIGD--PNLLLAIMGPKVTEYWTQGPAAEKASERLGSTE-RGQQLMAQHMTIFPTCSFLPGINTIRAWHPRGPNEIEVWAFTVV BedC1 ML2 215 QFCSDMYHAGTTAHLSGIIAGLPEDLELADLAPPKFGKQYRASWGGHGSGFYIGD--PNMMLAMMGPKVTSYLTEGPAAEKAAERLGSIE-RGTKIMLEHMTVFPTCSFLPGVNTIRTWHPRGPNEVEVWAFTVV BpdC1 M5 218 QFCSDMYHVGTTSHLSGLLAGLPDEIDIREVQPPTTGIQYSAPWGGHGSGFYIGE--MGTLAAVMGMKILEYYTSGPAAEKAAKRLGSAV-RGSQATGQHITVFPTCSFLPGINTIRTWHPRGPHEIEVWSFTVV TodC1 F1 215 QFCSDMYHAGTTSHLSGILAGLPEDLEMADLAPPTVGKQYRASWGGHGSGFYVGD--PNLMLAIMGPKVTSYWTEGPASEKAAERLGSVE-RGSKLMVEHMTVFPTCSFLPGINTVRTWHPRGPNEVEVWAFTVV CumA1 IPOl 227 QFCSDMYHAGTMAHLSGVLSSLPPEMDLSQVKLPSSGNQFRAKWGGHGTGWFNDD--FALLQAIMGPKVVDYWTKGPAAERAKERLGKV-LPADRMVAQHMTIFPTCSFLPGINTVRTWHPRGPNEIEVWSFIVV |
| *Figure S2: Multiple alignment of SVs of BphA sequences from the ^13^C-metagenome and their close homologues as shown in the phylogeny reconstruction in the Figure 2.* The percentage of abundance of the SVs in the dataset is indicated in round brackets. Regions III as discussed by Vezina et al. (2008) is highlighted by the grey background. Highly conserved regions are shown in red. Residues with greater than 50% identities are shown in blue. Abbreviation, source organism [Genbank accession no.]: ARHD LA-4, *Dyella ginsengisoli* LA-4 [WP_043599105.1]; BphA B-357, *Pseudomonas alcaligenes* B-357 [ABR08355.1]; ARHD W5, *Cupriavidus* sp. WS [WP_029044275.1]; BphA KF707, *Pseudomonas furukawaii* KF707 [BAU73321.1]; BphA LB400, *Paraburkholderia xenovorans* LB400 [AAB63425.1]; ARHD Gsoil 142*, Panacagrimonas perspica* Gsoil 142 [WP_133883168]; ARHD CCH12-A3, *Novosphingobium* sp. CCH12-A3 [WP_082734399.1]; BphA JB1, *Burkholderia* sp. JB1 [AJ010057]; BphA B-356, *Pandoraea pnomenusa* B-356 [AAC44526.1]; BphA KKS102, *Pseudomonas* sp. KKS102 [D17319]; BphA TK102, *Commamonas testosteronii* TK102 [BAC01052.1]; ARHD TR3.2, *Immundisolibacter cernigliae* TR3.2 [WP_068806964.1]; BphA P6, *Rhodococcus globerulus* P6 [CAA56346.1]; BphA RHA1, *Rhodococcus jostii* RHA1 [Q53122.1]; BedC1 ML2, *Pseudomonas putida* ML2 [Q07944]; BpdC1 M5, *Rhodococcus* sp. M5 [AAB07750.1]; TodC1 F1, *Pseudomonas putida* F1 [AAA26005.1]; CumA1 IP01, *Pseudomonas fluorescens* IPOl [BAA07074.1]. |

| 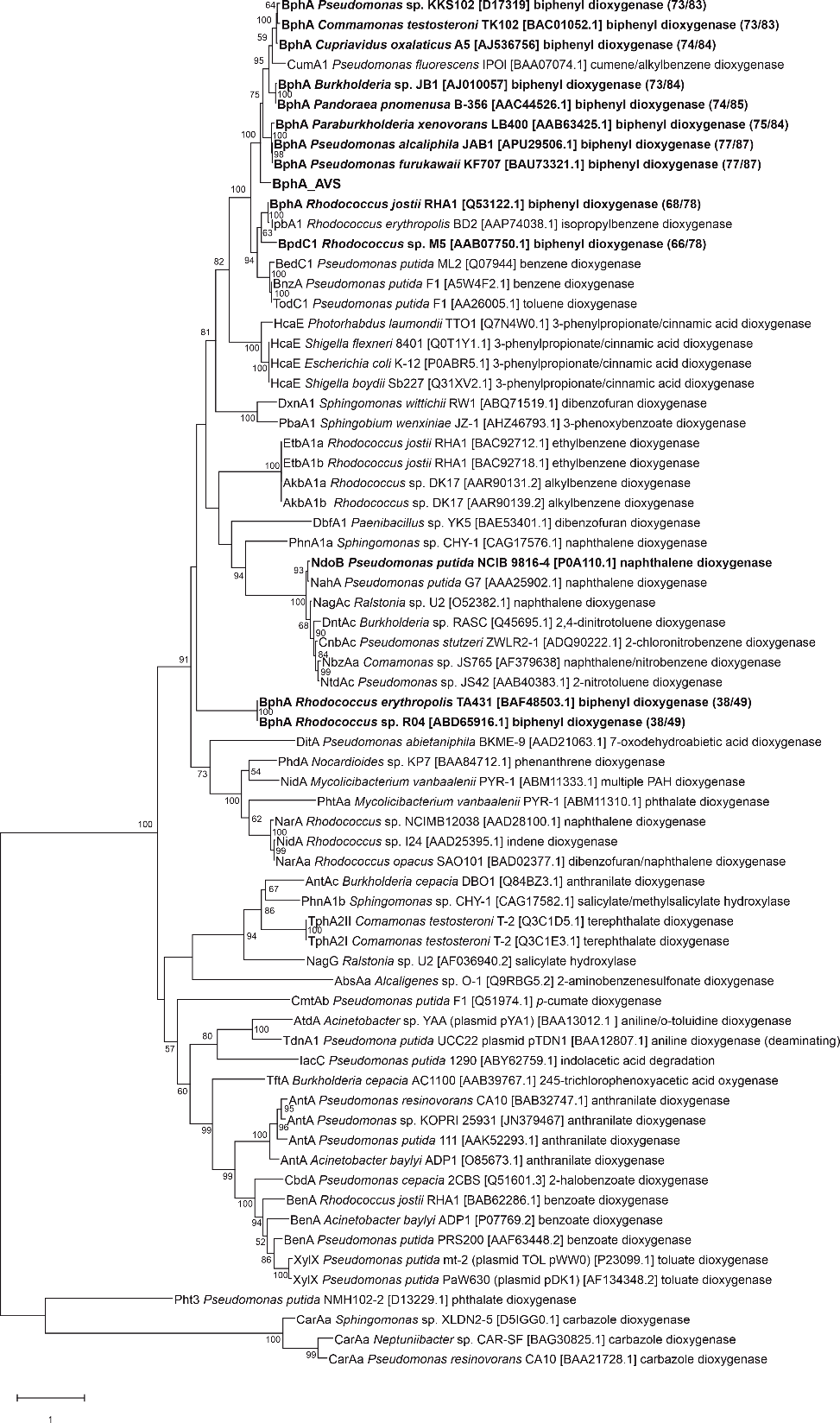 |
| --- |
| *Figure S3*: *Dendrogram showing position of BphA_AVS in the phylogeny reconstruction of exemplary ARHD large subunits.* Protein sequences the function of which was experimentally evidenced, were manually retrieved from Genbank (accession numbers shown in square brackets). Bootstrapping was used to test the tree topology (500 bootstraps), only the bootstrap values > 50 are shown. The tree is drawn to scale, branch lengths represent the number of substitutions per site. There were the total of 913 positions in the final dataset. The percent AA identity/similarity values with BphA_AVS of sequences discussed in the text (shown in bold) are indicated in round brackets. |

| *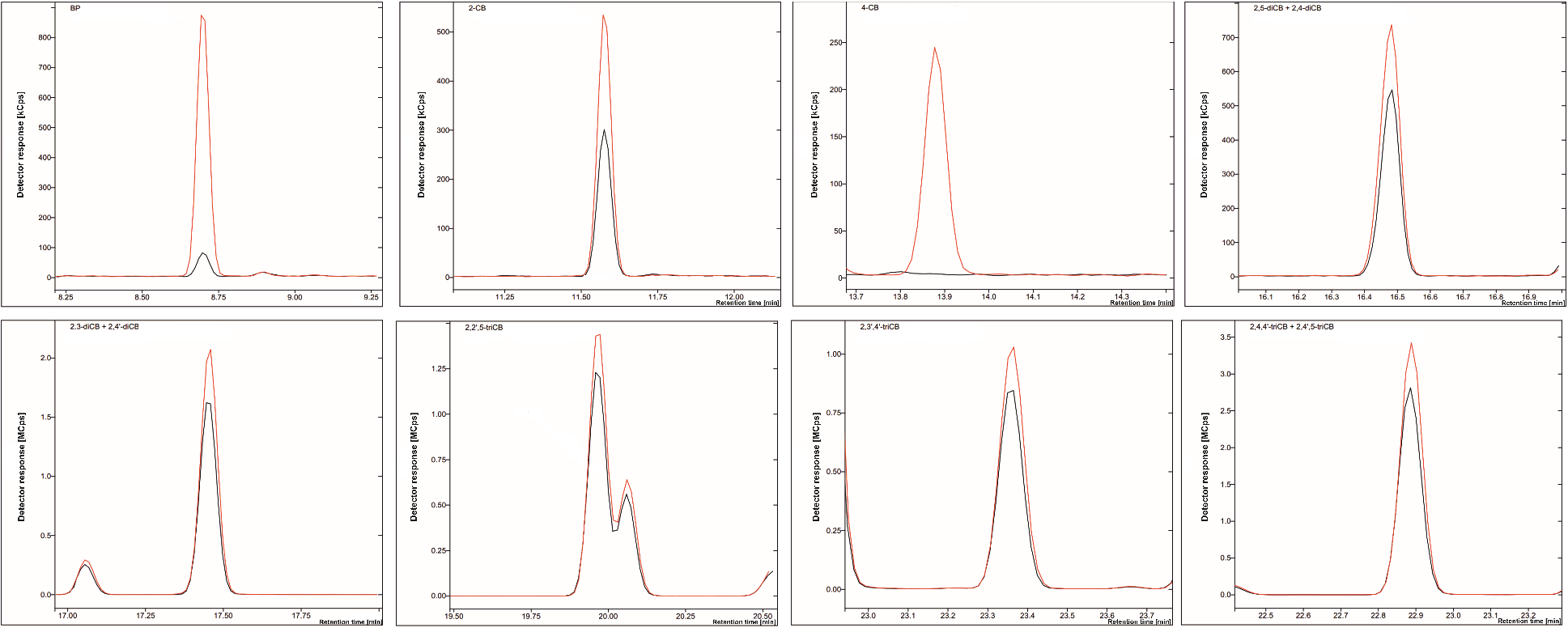* |
| --- |
| *Figure S4: Chromatograms of the PCB congeners depleted by* E. coli *cells expressing BPDO_AVS*. PCBs in the commercial mixture Delor 103 were analyzed using GC-MS (Garrido-Sanz et al., 2020). Cut-outs from the original chromatograms are shown due to the scale differences caused by the varying concentrations of the congeners in the mixture. |

*Table S1: Taxonomic assignment of ORFs identified in the scaffold bearing* bphA_AVS *according to NCBI and GTDB system.*

| NCBI Taxonomy | GTDB Taxonomy [Genus] | Number of ORFs | Average AA identity [%] |
| --- | --- | --- | --- |
| *Burkholderia* sp. *OLGA172* | *Paraburkholderia* | 11 | 97.1 |
| *Cupriavidus* |  | 7 | 67.0 |
| *Burkholderia insecticola* | *Caballeronia* | 6 | 84.2 |
| *Burkholderiales* |  | 4 | 74.3 |
| *Pseudomonas* |  | 4 | 68.0 |
| *Paraburkholderia aromaticivorans* | *Paraburkholderia* | 2 | 100.0 |
| *Paraburkholderia hospita* | *Paraburkholderia_A* | 2 | 97.4 |
| *Bacteria* |  | 2 | 86.2 |
| *Pandoraea oxalativorans* | *Pandoraea* | 2 | 85.8 |
| *Pseudoxanthomonas spadix* | *Pseudoxanthomonas_A* | 2 | 78.1 |
| *Cupriavidus malaysiensis* | *Cupriavidus* | 2 | 62.6 |
| *Thauera* sp. *K11* | *Thauera* | 2 | 62.2 |
| *Paraburkholderia fungorum* | *Paraburkholderia* | 1 | 92.6 |
| *Paraburkholderia* |  | 1 | 90.8 |
| *Cupriavidus necator* | *Cupriavidus* | 1 | 90.3 |
| *Pandoraea* |  | 1 | 83.6 |
| *Thiomonas intermedia* | *Thiomonas* | 1 | 79.6 |
| *Burkholderia cepacia complex* |  | 1 | 72.5 |
| *Burkholderia lata* | *Burkholderia* | 1 | 71.7 |
| *Paraburkholderia* sp. *SOS3* | *Paraburkholderia_A* | 1 | 70.9 |
| *Azospirillum* sp. *TSA2s* |  | 1 | 69.2 |
| *Thauera* sp. *MZ1T* | *Thauera* | 1 | 69.0 |
| *Cupriavidus* sp. *USMAHM13* | *Cupriavidus* | 1 | 63.5 |
| *unclassified Chelatococcus* |  | 1 | 63.3 |
| *Celeribacter indicus* | *Celeribacter* | 1 | 48.1 |

*Table S2: List of PCB congeners that were analyzed but not degraded by BPDO_AVS-bearing* E. coli *cells during resting cell assay.*

| Dichlorobiphenyls (diCB) | sum of 2,2'-diCB and 2,6-diCB, 2,3'-diCB, 4,4'-diCB |
| --- | --- |
| Trichlorobiphenyls  (triCB) | 2,2',3-triCB, 2,2',4-triCB, 2,2',6-triCB, 2,3,4'-triCB, sum of 2,3',4-triCB and 2,3',5-triCB, sum of 2,3,6-triCB and 2,3',6-triCB, 2,4',6-triCB, 3,4,4'-triCB |
| Tetrachlorobiphenyls  (tetraCB) | 2,2',3,3'-tetraCB, sum of 2,2',3,4-tetraCB and 2,3',4',6-tetraCB, 2,2',3,4'-tetraCB, 2,2',3,5'-tetraCB, 2,2',3,6-tetraCB, 2,2',3,6'-tetraCB, sum of 2,2',4,4'-tetraCB and 2,2',4,5-tetraCB, 2,2',4,5'-tetraCB, 2,2',4,6'-tetraCB, 2,2',5,5'-tetraCB, 2,2',5,6'-tetraCB, 2,3,3',4-tetraCB, sum of 2,3,3',4'-tetraCB and 2,3,4,4'-tetraCB, 2,3,4',5-tetraCB, sum of 2,3',4,4'-tetraCB and 2,3',4',5-tetraCB, 2,3',4,5-tetraCB, sum of 2,3',5,5'-tetraCB and 2,3,4',6-tetraCB, 2,4,4',5-tetraCB, 2,3',4',5'-tetraCB, 3,3',4,4'-tetraCB |
| Pentachlorobiphenyls  (pentaCB) | 2,2',3,3',4-pentaCB, sum of 2,2',3,3',6-pentaCB and 2,2',3,5,5'-pentaCB, 2,2',3,4,5'-pentaCB, 2,2',3,4',6-pentaCB, 2,2',3,5',6-pentaCB, 2,2',3,4',5'-pentaCB, 2,2',4,4',5-pentaCB, sum of 2,2',4,5,5'-pentaCB and 2,3,3',5',6-pentaCB, 2,3,3',4,4'-pentaCB, 2,3,3',4',6-pentaCB, 2,3,3',5,5'-pentaCB, 2,3',4,4',5-pentaCB |
| Hexachlorobiphenyls  (hexaCB) | sum of 2,2',3,4,4',5'-hexaCB and 2,3,3',4',5,6-hexaCB, 2,2',3,4',5,6'-hexaCB, 2,2',3,4',5',6-hexaCB, 2,2',4,4',5,5'-hexaCB |
| Heptachlorobiphenyl  (heptaCB) | 2,2',3,4,4',5,5'-Heptachlorobiphenyl |

References:

Garrido-Sanz, D., Sansegundo-Lobato, P., Redondo-Nieto, M., Suman, J., Cajthaml, T., Blanco-Romero, E., et al. (2020). Analysis of the biodegradative and adaptive potential of the novel polychlorinated biphenyl degrader Rhodococcus sp. WAY2 revealed by its complete genome sequence. *Microbial Genomics*. doi: <https://doi.org/10.1099/mgen.0.000363>.

Vezina, J., Barriault, D., and Sylvestre, M. (2008). Diversity of the C-terminal portion of the biphenyl dioxygenase large subunit. *J. Mol. Microbiol. Biotechnol.* 15(2-3)**,** 139-151.
